# Radiological Features of Human Papillomavirus (HPV)-Positive and HPV-Negative Oropharyngeal Squamous Cell Carcinoma (OPSCC)—Considerations for Multimodal Analysis

**DOI:** 10.3390/cancers18101648

**Published:** 2026-05-20

**Authors:** Nur Ayne Zaharoff, Oscar Emanuel, Umar Rehman, Shachi J. Sharma, Eleanor J. Crossley, Yuju Ahn, Winston Zhu, Jacklyn Liu, Dominic Wilkins, Jozsef Brunning, Claudia Kirsch, Jens Peter Klussmann, Timothy Beale, Matt Lechner, Simon Morley

**Affiliations:** 1 UCL Cancer Institute, University College London, London WC1E 6BT, UK; 2 Division of Surgery and Interventional Science, University College London, London W1W 7TH, UK; 3 Department of Radiology, Imperial College NHS Healthcare Trust, London W2 1NY, UK; 4 Department of Otorhinolaryngology, Head and Neck Surgery, Medical Faculty, University of Cologne, 50931 Cologne, Germany; 5 European Molecular Biology Laboratory, European Bioinformatics Institute (EMBL-EBI), Wellcome Genome Campus, Hinxton, Cambridge CB10 1SD, UK; 6 Department of Radiology, Yale School of Medicine, New Haven, CT 06520, USA; 7 Department of Radiology and Head and Neck Centre, University College London Hospitals NHS Trust, London NW1 2BU, UK

**Keywords:** human papillomavirus, imaging, oropharyngeal cancer

## Abstract

**Background:** Human papillomavirus (HPV)-positive oropharyngeal squamous cell carcinoma (OPSCC) differs from HPV-negative OPSCC in its molecular, biological, and clinical characteristics. We reviewed the literature on radiological differences between HPV-positive and HPV-negative OPSCC across ultrasound, CT, MRI, and PET-CT as this appears to be a critical gap in the literature. **Methods:** We performed a narrative review of studies reporting imaging findings in OPSCC by HPV status. Two reviewers independently searched PubMed, Ovid MEDLINE, Ovid EMBASE, and the Cochrane Library, from inception to February 2026, supplemented by searching major radiology journals and the grey literature. Eligible English-language studies included patients with OPSCC of known HPV status, assessed at least one imaging modality, and reported imaging findings stratified by HPV status. After title, abstract, and full-text screening, 66 studies were included. **Results:** HPV(+) OPSCC was more commonly associated with well-defined primary tumours and a higher prevalence of nodal metastases, particularly cystic nodal metastases and extranodal extension. These findings were broadly concordant with reported radiomic signatures. MRI studies suggested lower apparent diffusion coefficient values, while PET-CT studies suggested higher entropy and smaller primary lesions in HPV-positive disease. **Conclusions:** Selected imaging features may help distinguish HPV(+) from HPV(−) OPSCC, but current evidence remains insufficient for reliable standalone clinical application. Prospective multicentre validation and integration of multimodal imaging, radiomics, and radiogenomics are needed to improve non-invasive HPV stratification and support future precision diagnostics.

## Simple Summary

Oropharyngeal cancer caused by human papillomavirus (HPV) behaves differently from cancer that is not caused by this virus, and patients often have different outcomes. Although laboratory testing remains the standard way to confirm human papillomavirus status, medical imaging may also show patterns that help clinicians suspect this diagnosis earlier. In this review, we examined published studies comparing imaging findings in HPV-positive and HPV-negative oropharyngeal cancer across various types of scans. We found that HPV-positive tumours were more often linked to smaller primary tumours, cystic lymph node spread, and differences on advanced imaging measures. These findings suggest that imaging could support earlier suspicion of human papillomavirus-related disease and may, in the future, be combined with computational analysis to improve non-invasive tumour classification and guide more personalised care.

## 1. Introduction

Human papillomavirus (HPV) was first recognised as a causal factor in cervical cancer in the 1980s, but it was not until 2007 that the International Agency for Research on Cancer concluded that there was sufficient evidence to classify HPV as a cause of a subset of head and neck cancers [1,2,3]. Since then, the incidence of HPV-associated oropharyngeal squamous cell carcinoma (OPSCC) has risen substantially, particularly in high-income countries [4,5,6,7,8], to the extent that projected incidence of OPSCC in men is anticipated to exceed that of cervical cancer in women in high-income nations [6,7,8]. HPV(+) OPSCC is now recognised as a distinct clinicopathological entity from HPV-negative disease, with important differences in tumour biology, patient demographics, and clinical outcomes [9,10].

Although patients with HPV(+) and HPV-negative OPSCC may present with similar symptoms, the underlying mechanisms of carcinogenesis differ substantially. HPV-16 is the predominant viral subtype implicated in HPV-positive OPSCC, principally through the actions of the viral oncoproteins E6 and E7, which disrupt key tumour suppressor pathways [9]. Clinically, HPV(+) OPSCC more commonly affects younger patients, shows a strong male predominance, and often arises in individuals without substantial tobacco exposure [3]. It is also associated with greater sensitivity to chemoradiotherapy, improved survival, and lower rates of locoregional recurrence compared with HPV(−) disease [11].

Imaging has traditionally served as an adjunct to clinical examination in head and neck oncology, with established roles in pretreatment staging, post-treatment response assessment, and surveillance [12]. However, accumulating evidence suggests that imaging may also capture phenotypic differences associated with HPV status [13,14]. Conventional radiological features, as well as emerging quantitative approaches such as radiomics and radiogenomics, may help identify imaging patterns associated with HPV(+) disease. Although imaging cannot currently replace tissue-based diagnosis, it may contribute to earlier diagnostic suspicion, more focused tissue sampling, and improved risk stratification within a multimodal diagnostic framework [9,10,12].

Despite growing interest in imaging biomarkers for OPSCC, the radiological features that most consistently distinguish HPV(+) from HPV(−) disease across imaging modalities have not been clearly synthesised. The central question of this review is whether reproducible modality-specific imaging characteristics exist that may help differentiate HPV(+) from HPV(−) OPSCC and support non-invasive tumour stratification. We therefore undertook this narrative review to summarise reported imaging differences across ultrasound, computed tomography (CT), magnetic resonance imaging (MRI), and positron emission tomography-computed tomography (PET-CT), and to examine how these findings are informing emerging radiomic and radiogenomic approaches. We hypothesised that HPV(+) OPSCC demonstrates a distinct imaging phenotype that, while not replacing tissue diagnosis, may improve diagnostic suspicion, guide tissue sampling, and enhance risk stratification within multimodal clinical pathways.

## 2. Materials and Methods

### Search Strategy

We performed a literature search with a focus on identifying articles relating to imaging modalities in OPSCC. Two authors individually carried out an independent literature search of all records in PubMed, Ovid MEDLINE, Ovid EMBASE, The Cochrane Library, and ClinicalTrials.gov (accessed on 2 February 2026) from database inception until February 2026. The search was conducted in April 2025 and then an updated search was performed in February 2026. Any discrepancies or disagreements were resolved by a third independent reviewer. Both ‘free-text term’ and ‘MeSH term’ searches were completed, using variations of the keywords ‘head and neck cancer’/’HNSCC’/’oropharyngeal cancer’/‘OPSCC’ and ‘human papillomavirus’/’HPV’ and imaging/’radiology’. Only English language articles were considered.

The archives of major radiology journals, as well as grey literature and references from prior reviews, were searched for references, recent, or in-press articles that may have been missed by electronic searches. Each author’s results were merged, and duplicate citations were discarded. Articles describing the analysis of radiological findings in HPV(+) and HPV(−) OPSCC were included and are reviewed below. Following the screening of titles, abstracts, and full texts, a total of 66 articles were included in the narrative review (Supplementary Figure S1).

Studies were eligible for inclusion if they met the following criteria:

1. Included human participants with a diagnosis of OPSCC and known HPV status (HPV-positive or HPV-negative);
2. Evaluated at least one imaging modality—ultrasound (US), computed tomography (CT), magnetic resonance imaging (MRI), positron emission tomography–computed tomography (PET-CT)—or radiomics/radiogenomics applied to these modalities;
3. Reported imaging findings stratified by HPV status.

## 3. Results

A total of 66 articles were included in the narrative review. Of these, 6 examined ultrasound imaging [15,16,17,18,19,20], 7 focused on CT [13,14,21,22,23,24,25], 10 investigated PET-CT [21,26,27,28,29,30,31,32,33,34], 7 assessed MRI [11,35,36,37,38,39,40], 29 explored radiomics [16,41,42,43,44,45,46,47,48,49,50,51,52,53,54,55,56,57,58,59,60,61,62,63,64,65,66,67,68], and 10 addressed radiogenomics [16,69,70,71,72,73,74,75,76,77].

### 3.1. Imaging Modalities to Distinguish Between HPV+ and HPV− OPSCC

Various imaging techniques, including ultrasound, CT, PET-CT, MRI, radiomics, and radiogenomics, provide unique insights into the distinct anatomical, metabolic, and molecular characteristics of OPSCC. This section explores the radiological differences between HPV(+) and HPV(−) OPSCC across multiple modalities, highlighting their diagnostic value and emerging advancements in the field.

### 3.2. Ultrasound

Ultrasound contributes primarily to the evaluation of cervical nodal disease in HPV(+) OPSCC, where the most characteristic reported findings are a high prevalence of metastatic lymphadenopathy with cystic or necrotic change. Although these appearances are not specific to HPV-associated malignancy, they may provide an important early clue to the diagnosis, particularly in patients presenting with neck masses or cancer of unknown primary. In this context, ultrasound has an established role in nodal characterisation, image-guided tissue sampling, and, more recently, in treatment-response assessment imaging [15,16,17,18,19,20].

HPV(+) OPSCC has a greater predilection to nodal metastases than HPV(−), and those metastases are more likely to be cystic and necrotic, appearing hypoechoic to adjacent musculature on ultrasound [16]. It should be noted that cystic lymph nodes are not specific for HPV(+) OPSCC and can be seen in both tuberculosis and papillary thyroid cancer [17]. They should also not be mistaken for benign cystic disease of the neck, e.g., branchial cleft cyst or cystic hygroma. Different anatomical origins of head and neck cancer metastasise to specific nodal stations due to their drainage pathway, but there is no specific nodal site for HPV(+) OPSCC.

It should be noted that cystic or necrotic lymphadenopathy can reduce the sampling adequacy of fine needle aspiration due to its paucicellular nature, debris, and inflammatory material [18], and as such, it would be prudent to suspect HPV(+) OPSCC in nodes of that morphology with non-diagnostic cytology.

A recent study by Pellini et al. [20] has shown that ultrasound can also be useful to assess nodal response to chemoradiotherapy in OPSCC patients and offered quantification for the modality’s sensitivity to metastatic disease; its use as a standalone modality is limited for the detection of metastatic disease given its sensitivity of 77.8%, but its concurrent use with MRI and PET-CT produced a specificity and positive predictive value of 100%. Furthermore, ultrasound scanning is regarded as a cost-effective, readily available, and radiation-free method to allow the characterization of OPSCCs by providing better direct visualization of structures outside the traditional orthogonal planes of the body imaged by CT, MRI, and PET-CT. Relatively new ultrasound techniques such as endoscopic ultrasound [19] have also enabled investigation of abnormalities in the tonsils and the base of the tongue, which can potentially aid in identifying the primary sites of head and neck cancers of unknown primaries [15].

### 3.3. CT

CT literature suggests that HPV(+) OPSCC demonstrates a distinct radiological pattern compared with HPV(−) disease, characterised by a tendency to arise from the tonsillar and base of tongue lymphoepithelium, a greater likelihood of occult primary presentation, and a higher prevalence of cystic or low-attenuation nodal metastases. In contrast, HPV(−) tumours are more often described as ill-defined primary lesions with greater invasion of adjacent structures. Emerging quantitative CT data also suggest that p16-positive primary tumours and nodal metastases may have lower attenuation than p16-negative disease, supporting the concept of a recognisable HPV-associated imaging phenotype [13,14,21,22,23,24,25].

The literature broadly reports similar nodal findings on CT and ultrasound. Since Goldenberg et al. published their findings in 2007 [10], more current studies have also reported different radiological features between HPV(+) and HPV(−) OPSCC, particularly within its nodal metastasis [13]. Goldenberg et al. found that 87% of the 20 patients with cystic nodal metastases on radiological review of CT or MRI films were found to have HPV DNA within the lymph node metastases [13]. The cystic nodes were described to be well-circumscribed and commonly surrounded by a smooth fibrous capsule and filled with a variation between straw-coloured to thick brown fluid on aspiration. In a matched and blinded retrospective review of CT images, Cantrell et al. also found significantly more cystic nodal metastases in HPV(+) OPSCC [22].

In a recent retrospective review, Fujita et al. reported a higher prevalence of nodal metastases and extracapsular spread in HPV(+) patients compared with HPV(−) patients [23]. Although no cystic nodal metastases were reported in HPV(−) patients, the study reported cystic nodal changes in only 16% of its HPV(+) cohort, suggesting limitations in placing undue emphasis on cystic nodes as the primary imaging indicator of HPV(+) OPSCC. HPV(+) OPSCC nodal metastases can be seen in the deep cervical chain, with the metastasis showing central necrosis manifesting as reduced enhancement (Figure 1). The study also described differences in the radiological features of the HPV(+) and HPV(−) primary tumours. It was reported that HPV(−) primary tumours more frequently had ill-defined borders and increased invasion of adjacent muscles.

The Hounsfield unit (HU) is a quantitative measurement of radiodensity used in CT imaging [21]. Suto et al. demonstrated that the attenuation of unenhanced CT images of the solid component within HPV(+) nodal metastases was significantly lower in patients with recurrence compared to those without (44.1 HU vs. 47.1 HU, *p* = 0.010) [24]. Similarly, in HPV(−) nodal metastases, the attenuation on contrast-enhanced CT of the solid component was also significantly lower in patients with recurrence compared to those without (79.4 HU vs. 87.9 HU, *p* < 0.05) [24].

In addition to qualitative differences in nodal morphology, CT may also demonstrate quantitative differences between p16-positive and p16-negative OPSCC. One study found that p16-positive primary tumours and metastatic lymph nodes had significantly lower mean attenuation than p16-negative tumours (78.6 vs. 96.0 HU and 38.8 vs. 88.7 HU, respectively; both *p* < 0.0001), supporting the association of p16-positive disease with cystic, low-attenuation nodal metastases [25].

### 3.4. PET-CT

PET-CT literature suggests that HPV(+) OPSCC may demonstrate a distinct, though somewhat heterogeneous, metabolic imaging profile compared with HPV(−) disease. Reported differences include smaller primary tumour metabolic volumes, variation in measures of tumour heterogeneity and entropy, and characteristic nodal metastatic features that parallel the cystic and necrotic appearances seen on CT and ultrasound. PET-CT is also clinically important in HPV(+) disease because of its sensitivity for nodal and distant metastatic assessment, including the identification of unusual metastatic patterns and later distant recurrence. Taken together, these findings support a contributory role for PET-CT in non-invasive HPV stratification, while also underscoring the need for further validation of quantitative PET-derived biomarkers [21,26,27,28,29,30,31,32,33,34].

Current studies have shown two distinguishing factors that have potential as independent markers of HPV status in OPSCC: tumour heterogeneity and tumour median total volume (MTV). Cheng et al. extracted tumour uniformity data from FDG-PET-CT images using a normalised gray-level co-occurrence matrix, reporting lower tumour uniformity (or higher tumour heterogeneity) and higher entropy in HPV(+) patients [27]. In contrast to this, another study found that HPV(−) primary tumours are more heterogeneous than HPV(+) primaries whilst HPV(+)lymph nodes, which tend to be more cystic, are more heterogeneous than their counterparts [28]. In a study by Tahari et al., heterogeneity was calculated as the ratio of SUV max and SUV mean [28]. Sharma et al. reported a significantly higher intraindividual homogeneity of FDG-uptake in the primary lesion when compared to the respective lymph nodes in HPV(+) patients compared to HPV(−) patients [29].

Furthermore, Tahari et al. found that HPV(−) primary lesions are significantly larger than HPV(+) primary lesions, with a tumour MTV of 21.9 and 8.5, respectively [28]. Higher tumour MTV is associated with a significant increased risk of loco-regional recurrence, distant metastases, disease progression, and death [30,31,32]. Contradictory to these findings, Sharma et al. did not find a difference of MTV in HPV(+) versus HPV(−) tumours and showed that patients with PET-parameters above the respective median of their cohort had a significantly worse 5-year overall survival, regardless of HPV status [29]. For a representative example, please refer to Figure 2.

Again, nodal metastases are a helpful differentiator between HPV(+) and HPV(−) OPSCC, with contrast-enhanced ^18^F-FDG PET-CT being more sensitive for HPV(+) OPSCC than non-enhanced [21]. Its negative predictive value of 100% in 77 HPV(+) OPSCC cases reported by Chan et al. is very useful in obviating dissection [33].

Within the context of FDG-avid distant metastatic disease, there are significant differences depending on HPV status. The differences lie not in the incidence of distant metastasis but in their location, with HPV(+) OPSCC able to disseminate to multiple organs and unusual locations, and also in their clinical behaviour, with later manifestation around 3 to 5 years post-treatment completion [28,29,30,31,32,33].

Although HPV(+) OPSCC is classically associated with cystic nodal metastases and relatively small primary tumours, Kaka et al. identified a highly aggressive subset of HPV(+) OPSCC distinct radiologic features, including frequent cystic and necrotic nodal change, extracapsular spread, nodal clustering/matted nodes, larger nodal volumes, and high metabolic activity on FDG-PET [34]. In this cohort, all cases with lymphadenopathy demonstrated nodal clustering and extracapsular spread, and most showed cystic/necrotic nodal change. Two tumours also demonstrated a small-cell component pathologically, suggesting that a minority of HPV(+) tumours may follow a more aggressive radiologic–pathologic phenotype rather than the more typical favourable imaging pattern.

### 3.5. MRI

MRI has an important role in distinguishing HPV(+) from HPV(−) OPSCC because of its superior soft-tissue resolution and its capacity to provide quantitative diffusion-based imaging biomarkers. Across the available literature, HPV(+) tumours tend to present with lower T stage and lower ADC values within the primary lesion than HPV(−) tumours, suggesting differences in tissue microstructure that may relate to treatment responsiveness and tumour biology. At the same time, interpretation is complicated by the frequent presence of cystic nodal metastases in HPV(+) disease, which may demonstrate increased diffusion and therefore higher apparent diffusion coefficients (ADC) values than the primary tumour. Taken together, these findings support MRI, and particularly diffusion-weighted imaging, as a promising modality for non-invasive HPV characterisation, although standardisation and further validation remain necessary [11,35,36,37,38,39,40].

Recent studies using MRI have focused on the correlation between HPV status in OPSCC and the apparent diffusion coefficients (ADCs) of tumours. The ADC is a measure of the diffusion of water molecules within tissue and is commonly calculated using diffusion-weighted MRI. Nakahira et al. found significantly lower ADC values in MRI images of HPV(+) patients, with a mean ADC cut-off value of 1.027 × 10^−3^ mm^2^/s for HPV(+) tumours, yielding a sensitivity of 83.33% and a specificity of 78.57% [36]. Similarly, a retrospective study by Driessen et al. also found lower ADCs in HPV(+) patients [37]. This association was further supported by a study showing that mean ADC had the strongest discriminatory value among MRI parameters, and that combining ADC with smoking status improved classification of HPV status, achieving an AUC of 0.944, with 83.3% sensitivity and 92.6% specificity [40].

The presence of cystic change within nodes leads to increased diffusion due to the presence of free-moving fluid, with both tumour cellularity and water content reflected by its ADC values. We have established during this article that HPV(+) nodal metastases are more likely to be cystic; it is worth pointing out that these nodal ADC values will be higher despite the aforementioned studies finding lower ADC values in the primary tumour. For these reasons, the ADC values of the primary tumour and (especially cystic) nodes may well be very different, and comparing the mean ADC in HPV(−) and HPV(+) cases is therefore challenging. A lower ADC value in solid components of the tumour may indicate less necrosis, hypoxia, and acidosis; this may potentially explain why HPV(+) OPSCC is more responsive to chemoradiotherapy [38]. In reality, there are multiple reasons, both positive and negative, for the patients’ prognosis. For example, a low ADC value due to the tumour being less necrotic has a different clinical relevance to a low ADC value due to tumour hypercellularity.

Prior work has also found mean ADC was lower and skewness was higher in HPV(+) compared to HPV(−) OPSCC [39]. However, these findings were dependent on the b-values, which represent a parameter used to quantify the degree of diffusion weighting in the images. The ADC b0-1000 map was identified as the best method for distinguishing between HPV(+) and HPV(−) OPSCC.

In summary, the above studies are somewhat contradictory but show a potential value of ADCs in solid components of the tumour as a quantitative for HPV status in OPSCC. As discussed, it is important to understand that ADC values in tumours need to be taken in context, as there are multiple reasons for them being high or low. For representative examples, please refer to Figure 3, Figure 4, Figure 5 and Figure 6.

### 3.6. Radiomics

Radiomics offers a promising non-invasive approach to HPV stratification in OPSCC, with early studies showing moderate predictive performance and suggesting that HPV(+) tumours may be more compact and homogeneous than HPV(−) disease. However, current evidence remains limited by small study sizes and requires further validation [16,41,42,43,44,45,46,47,48,49,50,51,52,53,54,55,56,57,58,59,60,61,62,63,64,65,66,67,68].

Zhu et al. were able to predict HPV status by radiomics with an area under the receiver operating characteristic curve (AUCs) of 0.71, which suggests an acceptable discrimination in HPV status [42]. Bogowicz et al. found similar results for prediction accuracy (AUC = 0.78) [43]. Huang et al. were also able to show a significant ability to distinguish HPV(+) from HPV(−) tumours (AUC = 0.73) and specified that the dominant features were wavelet features followed by shape and size features and first-order features (which are calculated directly from the intensity values, including features such as minimum, maximum, mean, and variance) [44]. Haider et al. explored PET-CT radiomics for risk stratification in HPV-associated OPSCC in relation to progression-free and overall survival [45]. They found that radiomic data provides complementary information to the current American Joint Committee on Cancer (AJCC) staging scheme in a cohort of 311 patients. Their survival models relying on radiomics predictors alone outperformed AJCC baseline models in the majority of permutations [45].

However, caution is warranted when interpreting these radiomics studies, as most have relatively small sample sizes (<150), with the exception of the study by Haider et al. [45]. Nonetheless, the observed predictive performance highlights the potential of imaging for assessing tumour molecular status in the future.

Recent advances in CT-based radiomics suggest that habitat-level analysis may further improve HPV prediction. In contrast to whole-tumour radiomics, habitat radiomics subdivides the tumour into spatially distinct subregions and may therefore better capture intratumoral heterogeneity [47]. In one study, a habitat radiomics classifier outperformed intratumoral and combined radiomics models for HPV classification, while also identifying imaging characteristics of HPV(+) tumours as more compact and homogeneous and HPV(−) tumours as more irregular and heterogeneous [47]. These findings suggest that radiomics may offer not only predictive value but also insight into biologically meaningful imaging phenotypes within OPSCC.

HPV ascertainment and study methodology were highly heterogeneous across the literature. While some studies used confirmatory HPV-specific testing alongside p16 immunohistochemistry in keeping with guideline recommendations, many relied on p16 alone as a surrogate marker, and some did not clearly report the testing method. There was also marked variation in imaging modality, volume of interest, and model development, with studies using CT, MRI, CE-CT, or 18F-FDG PET and deriving features from primary tumours, nodal disease, or combined regions [16,41,42,43,44,45,46,47,48,49,50,51,52,53,54,55,56,57,58,59,60,61,62,63,64,65,66,67,68].

### 3.7. Radiogenomics

Radiogenomics extends radiomics by linking imaging phenotypes to underlying molecular and genomic characteristics, with the aim of improving biological tumour characterisation and ultimately supporting more personalised diagnosis and treatment. Early studies in OPSCC and broader HNSCC suggest potential associations between imaging heterogeneity and specific genetic or epigenetic alterations, but the evidence remains preliminary and based on small cohorts. At present, radiogenomics is best viewed as a promising but early-stage field with potential to refine non-invasive tumour stratification in the future [16,69,70,71,72,73,74,75,76,77].

Zwirner et al. investigated a cohort of 20 HNSCC patients, 14 of whom had OPSCC, to identify mutated driver genes [73]. Their results validated the established somatic mutation profiles of the Cancer Genome Atlas Network in 2015 [16]. *TP53*, *FAT1*, and *KMTD2* were the most frequently mutated driver genes, with only the *FAT1* mutation, seen predominately in the HPV(−) cases, displaying any correlation with radiomic features of heterogeneity, particularly reduced radiomic intra-tumour heterogeneity, and better survival outcomes. Again, using panel-sequencing on 327 genes as Zwirner et al. did, Clasen et al. studied intrapatient heterogeneity through spatial sampling and functional imaging by FDG-PET-MRI in 6 patients with OPSCC, 4 of whom were determined to be negative for HPV (P16 surrogate marker negative) and 2 who were not tested for HPV [73,74]. The genetic heterogeneity of the primary tumours was limited, with driver gene mutations found ubiquitously leading them to conclude that they were unable to identify a conclusive correlation between genetic heterogeneity and heterogeneity of the PET-MRI-derived parameters.

HPV(+) OPSCC was found by Katsoulakis et al. to have higher DNA methylation levels compared to non-HPV-related HNSCC and normal controls [75], building on prior research that suggested the likely driving difference in methylation clustering is HPV(+) tumours differentiating CpG island methylation. Katsoulakis et al. speculate that radiomics may capture methylation status as epigenetic factors make ideal therapeutic targets. Despite its relative infancy, the field of radiogenomics is certainly an exciting prospect in cancer diagnosis and treatment of the future [75].

More recent MRI-based radiogenomic studies in OPSCC have highlighted the importance of methodological optimisation for model generalisability. In one multicentre study, particle swarm optimisation (PSO)—a computational method that searches large numbers of possible feature combinations—combined with adaptive LASSO and Shapley values—methods used to refine feature selection and estimate the contribution of individual variables—achieved more consistent external validation performance for HPV status prediction than other feature-selection approaches [76]. Similarly, a separate study found that image post-processing steps, including harmonisation of data acquired across centres, removal of unstable features, and exclusion of highly correlated variables, substantially improved the external performance of MRI-based HPV prediction models [77]. Together, these findings suggest that careful preprocessing and feature-selection methods are important determinants of robustness and transferability in radiogenomic models.

Radiological findings by modality in OPSCC patients that are HPV(+) versus HPV(−) are summarised in Table 1. Findings regarding tumour heterogeneity and related quantitative imaging biomarkers were not fully consistent across studies or imaging modality. These conflicting and context-dependent findings are summarised in Table 2.

## 4. Discussion

This review synthesises the available literature on imaging features that may assist in distinguishing HPV(+) from HPV(−) OPSCC (Table 1). Across modalities, several recurring patterns emerge. HPV(+) disease is more often associated with smaller or occult primary tumours, greater nodal burden, cystic or necrotic nodal metastases, and lower ADC values in the solid components of the primary tumour, whereas HPV(−) tumours more commonly present as larger, more infiltrative primaries with less characteristic nodal morphology [13,14,15,16,20,22,28,29,30,36,37,39,46,78]. However, although these associations are clinically informative, the evidence remains heterogeneous and at times contradictory. No single imaging feature or modality currently provides sufficient accuracy to discriminate reliably between HPV(+) and HPV(−) OPSCC in routine clinical practice [13,14,15,16,20,22,28,29,30,36,37,39,46,78]. By contrast, radiomics appears more promising, with several studies reporting meaningful predictive performance for HPV status, although these findings remain preliminary and require validation in larger cohorts [42,43,44,45].

These limitations are reflected in the variability of the published literature. Imaging alone is susceptible to false-positive interpretation, with rates reported to be as high as 25%, even with PET-CT [79]. Similar concerns apply to other modalities, including MRI [80,81]. The findings summarised in this review therefore support a role for imaging in raising diagnostic suspicion and informing targeted investigation, rather than replacing tissue confirmation. In clinical practice, imaging must remain integrated with histopathology and molecular testing for accurate classification of HPV-related disease. Photon-counting CT is an emerging technology with potential relevance to head and neck oncology because it offers higher spatial resolution, reduced artefacts, and virtual monoenergetic image reconstruction; however, further work is needed to define its value specifically in HPV(+) and HPV(−) OPSCC [82,83].

The most consistent contribution of conventional imaging lies in pattern recognition rather than definitive classification. Ultrasound and CT repeatedly identify cystic or necrotic nodal metastases as characteristic, though not specific, features of HPV(+) disease, while MRI studies suggest that lower ADC values in the primary tumour may represent a more reproducible quantitative correlate of HPV positivity [36,37,38]. MRI offers superior soft-tissue contrast to better delineate adjacent structures such as nerves for perineural spread, diffusion-weighted imaging that allows for assessment of proton mobility, as well as blood flow perfusion, via such techniques as arterial spin labelling [84]. These techniques may enable more precise evaluation of both primary tumour, adjacent tissue involvement including bones, nerves, and nodal disease in relation to HPV status and require further investigation [60].

PET-CT findings are more variable, particularly with respect to tumour heterogeneity and metabolic tumour volume, likely reflecting methodological differences in feature extraction and patient selection [27,28,29]. The results also suggest that HPV(+) OPSCC is not radiologically uniform. In addition to the more typical phenotype of smaller primaries and cystic nodal disease, a minority of HPV(+) tumour subtypes may follow a more aggressive radiologic–pathologic course, including larger nodal volume, extracapsular spread, nodal clustering, and high metabolic activity. This heterogeneity is important, as it cautions against oversimplified interpretation of HPV-associated imaging features [34].

More recently, attention has shifted towards artificial intelligence (AI), including machine learning and deep learning (DL), as potential tools to enhance radiological interpretation and improve clinical decision making [85,86,87]. DL-based radiomics can identify intratumoural properties that are not appreciable on conventional visual assessment and may therefore offer a more sensitive means of capturing biological differences between HPV(+) and HPV(−) tumours. In particular, habitat-based radiomics appears capable of capturing spatially resolved intratumoural heterogeneity beyond whole-tumour feature extraction [47]. Such approaches may prove especially relevant in OPSCC, where the present review suggests that conventional morphology alone does not fully account for the biological diversity of HPV-related disease [47].

Molecular studies also indicate that HPV(+) disease comprises biologically distinct subgroups. A key limitation of current imaging and radiomics studies is that they infer tumour biology indirectly, without resolving the cellular heterogeneity that may underlie HPV-associated imaging phenotypes. Future work should therefore integrate radiomics and radiogenomics with single-cell and spatial multi-omics to determine whether features such as cystic nodal disease, diffusion restriction, metabolic heterogeneity, or radiomic habitats correspond to specific malignant cell states, viral transcriptional programmes, immune-cell populations, or stromal architectures. Although not OPSCC-specific, recent studies in hepatocellular carcinoma and melanoma demonstrate how single-cell sequencing integrated with bulk transcriptomics and machine learning can identify immune-cell populations and gene signatures associated with immunotherapy response, providing a useful methodological precedent for HPV(+) OPSCC research [88,89].

Transcriptomic analyses have identified separate HPV(+) subclasses with differing viral integration patterns, genomic alterations, immune signalling, and radiosensitivity, including subgroups associated with improved outcomes [90,91]. Although not defined by imaging alone, these findings provide a biological rationale for radiogenomic approaches that combine imaging with molecular profiling to better stratify HPV(+) OPSCC and potentially inform treatment intensity [90,91].

The integration of AI-enhanced radiomics and radiogenomics into OPSCC diagnostics may ultimately offer a more precise and potentially cost-effective approach to tumour characterisation [92]. Radiogenomics may provide an alternative to broad genomic sequencing, which can be constrained by sampling limitations, tumour heterogeneity, and cost [93,94,95]. Evidence from other tumour types, including BRCA testing in breast cancer and prognostic modelling in glioma, suggests that radiogenomic models can yield clinically meaningful gains with relatively modest incremental cost, particularly as sequencing costs continue to fall [93,94,95]. Nonetheless, implementation in OPSCC would require investment in software, infrastructure, and specialist training, and prospective health–economic studies will be needed to determine cost-effectiveness, scalability, and long-term clinical benefit across healthcare settings.

Radiomic deep learning models have already been applied in other head and neck cancers to predict recurrence-free, disease-free, and overall survival. Future work in OPSCC may integrate these approaches with tissue-level genomic and biomarker analysis [85,86]. In addition, combination with serum HPV DNA and E6/E7 serology, which have been associated with improved survival outcomes in OPSCC [87], could support a more comprehensive multimodal framework for diagnosis, prognostication, and treatment stratification. Circulating tumour HPV-DNA (ctHPV-DNA) represents a promising complementary biomarker in HPV(+) OPSCC for diagnosis, prognosis, early detection of recurrence, and treatment surveillance [96,97]. Available evidence suggests that ctHPV-DNA levels correlate with radiological tumour burden, that rapid clearance is associated with favourable radiological response, and that follow-up ctHPV-DNA levels may correspond with imaging-based treatment evaluation. While these observations are encouraging, further work is required to determine how ctHPV-DNA kinetics can be integrated into routine practice. Future trials should evaluate ctHPV-DNA alongside radiological assessment, particularly to clarify equivocal findings and to improve discrimination between true and false-positive radiological appearances [96,97].

From a clinical perspective, imaging findings suspicious for HPV(+) OPSCC, particularly cystic nodal metastases in the absence of substantial smoking history, should prompt targeted fine-needle aspiration with p16/HPV testing. Where available, serum HPV DNA or E6/E7 antibody testing may provide complementary information [98]. Interpretation of these data is likely to be most effective within a multidisciplinary setting, where imaging, pathology, and molecular findings can be integrated to guide management. The goal is not for imaging to replace biopsy, which remains the gold standard investigation, but to supplement diagnosis. Improved non-invasive characterisation of HPV status via imaging alongside biopsy information may have practical implications not only for diagnosis but also for prognostication, patient counselling, and selection into trials evaluating risk-adapted therapy. This is particularly relevant in HPV(+) OPSCC, where interest in treatment de-escalation continues, but where better tools are needed to distinguish more favourable from more aggressive biological subgroups [99].

Future research should prioritise prospective, multicentre cohorts with standardised imaging protocols to improve reproducibility and reduce methodological heterogeneity. Radiomic models require rigorous external validation in independent populations before they can be considered clinically generalisable. Integrative studies combining imaging with tissue-based genomic profiling and circulating biomarkers, such as HPV DNA or E6/E7 antibody serology, may help define more precise multimodal diagnostic pathways [98]. Interventional studies are also needed to determine whether earlier radiological suspicion of HPV(+) disease can shorten time to diagnosis or treatment and improve clinical outcomes. Finally, broader inclusion of geographically and demographically diverse populations will be essential to ensure global applicability and to avoid widening disparities in diagnostic innovation. Although not yet ready for routine clinical implementation, radiomics and radiogenomics are likely to play an increasingly important future role in OPSCC by enabling non-invasive phenotyping, improving biological stratification, and supporting multimodal decision making alongside pathology and circulating biomarkers. Their greatest potential may lie not in replacing tissue diagnosis but in augmenting risk prediction, refining patient selection for biopsy and clinical trials, and identifying imaging signatures linked to treatment response and outcome.

Figure 7 summarises a potential diagnostic pathway based on the current evidence. In this model, first-line imaging with ultrasound, MRI, CT, or PET-CT identifies features suggestive of HPV-related disease and prompts targeted tissue sampling with p16/HPV testing. Where available, serological assays such as plasma HPV DNA and E6/E7 antibody testing may further refine diagnosis and prognostication. Although current implementation still depends heavily on radiologist expertise, future integration of machine learning-based radiomics and radiogenomics could support automated identification of suspicious imaging signatures, prioritisation for biopsy, and more efficient multidisciplinary decision making. In resource-limited settings, the cost-effectiveness of introducing advanced imaging and AI-supported analysis will require formal evaluation, balancing potential diagnostic gains against the costs of technology, training, and infrastructure.

This review should be interpreted in light of several limitations. The available evidence is heterogeneous, with substantial variation in study design, sample size, imaging protocols, HPV ascertainment methods, and quantitative image analysis. Furthermore, many of the included studies were retrospective and single-centre, limiting generalisability and increasing the risk of selection bias. Direct comparison across modalities is challenging because reported imaging end points were inconsistent, ranging from qualitative morphological descriptors to quantitative parameters such as ADC, SUV-derived heterogeneity metrics, and radiomic features. Several radiomics and radiogenomics studies were based on relatively small cohorts and lacked external validation, raising the possibility of overfitting and limiting immediate clinical applicability. Moreover, some studies used p16 as a surrogate marker for HPV status rather than direct viral testing, which may introduce misclassification and further contribute to inconsistency across the literature. Finally, most studies lack paired single-cell or spatial molecular data, limiting mechanistic interpretation of how macroscopic imaging features relate to cellular heterogeneity, immune microenvironment, and HPV-driven tumour biology.

## 5. Conclusions

Taken together, the available evidence suggests that imaging can contribute meaningfully to the diagnostic and prognostic evaluation of OPSCC, but not yet to definitive determination of HPV status. Histological and molecular confirmation remain the standard, while radiological features provide complementary information that may raise suspicion for HPV-related disease and help refine subsequent investigation. The most reproducible findings across the literature include cystic nodal metastases, lower attenuation nodal disease on CT, lower primary tumour ADC on MRI, and, in emerging radiomics studies, more compact and homogeneous phenotypes in HPV(+) tumours. The convergence of conventional imaging, advanced computational analysis, and molecular profiling represents a promising direction in head and neck oncology.

Radiomics and radiogenomics are unlikely to replace histological and molecular confirmation in the near term, but they have clear potential to become clinically useful adjuncts in OPSCC by enabling more refined non-invasive tumour phenotyping and biomarker-informed stratification. Their future role will depend on successful validation in large multicentre datasets, integration with tissue and liquid biomarkers, and demonstration that AI-supported models can improve diagnosis, prognostication, and treatment planning in a reproducible and clinically meaningful way.

## Figures and Tables

**Figure 1:**
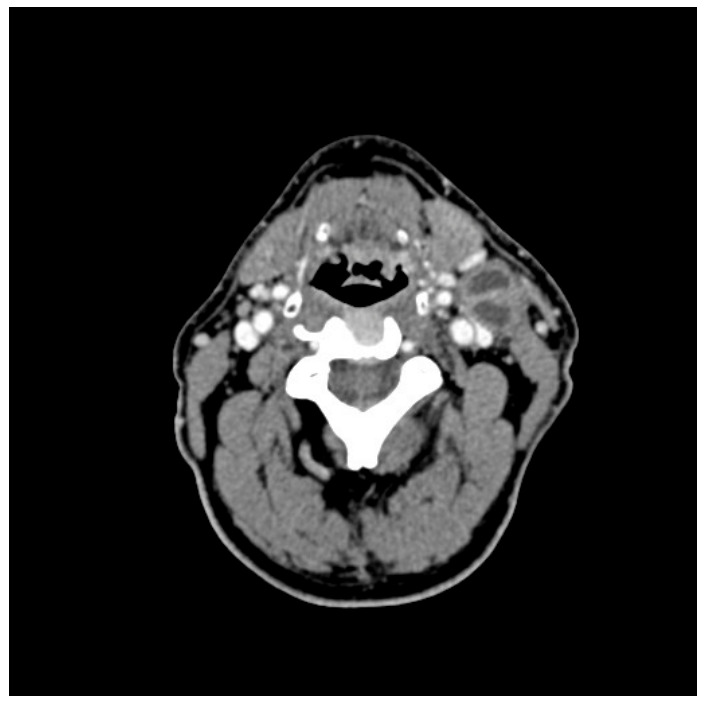
An axial contrast-enhanced CT demonstrating HPV(+) OPSCC nodal metastasis in the left deep cervical chain. The metastasis shows central necrosis manifesting as reduced enhancement.

**Figure 2:**
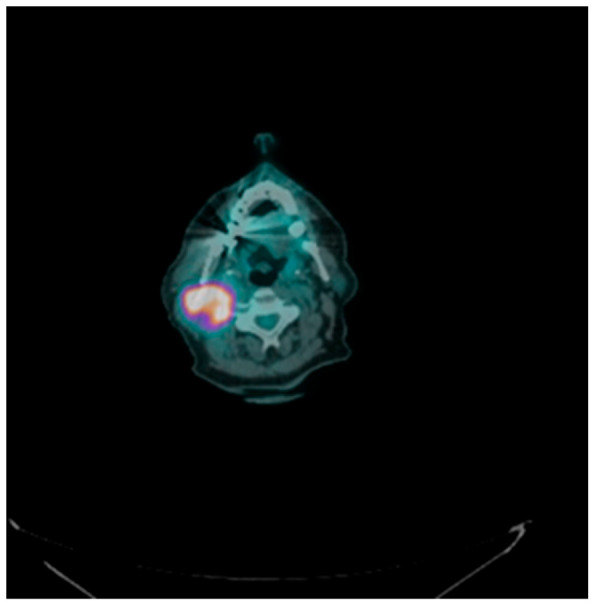
An axial PET-CT in an HPV(−) patient. Right nodal mass in the right upper deep cervical chain demonstrating high heterogeneity.

**Figure 3:**
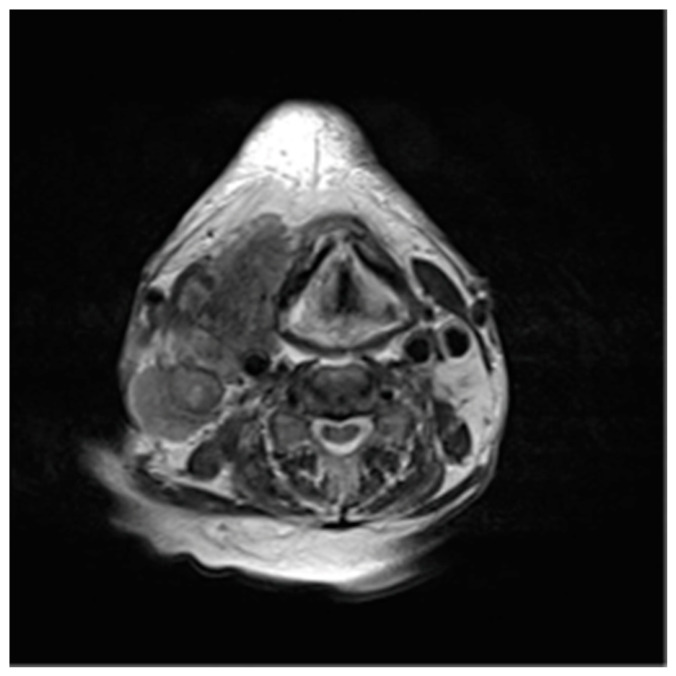
An axial MRI showing a right-sided metastatic nodal mass in an HPV(−) patient. The nodal mass is large, solid (with no cystic change), and demonstrates extracapsular spread and intramuscular invasion.

**Figure 4:**
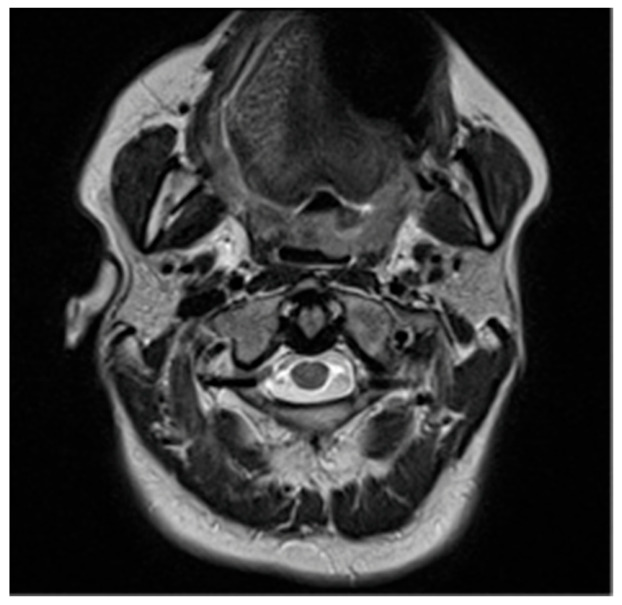
An axial MRI demonstrating an HPV(+) patient with a small volume left oropharyngeal (tonsillar) primary tumour confined to the tonsillar fossa.

**Figure 5:**
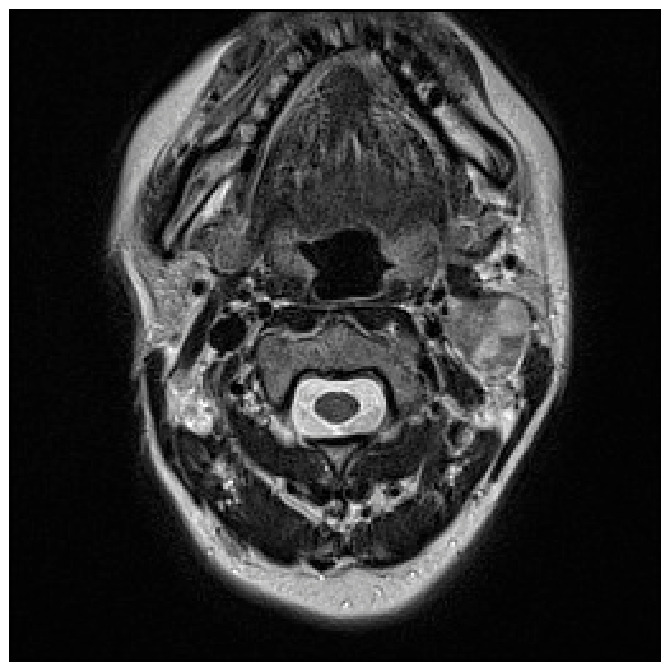
An axial MRI demonstrating left cystic (high T2 signal) deep cervical chain nodal metastatic disease in an HPV(+) patient.

**Figure 6:**
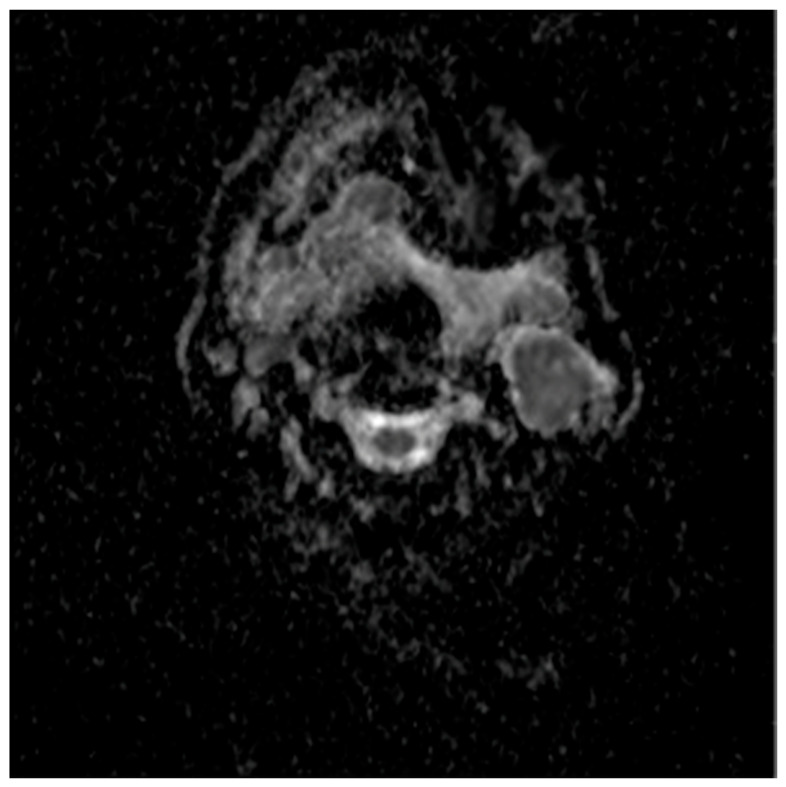
ADC Map MRI showing restricted diffusion in the left neck nodal metastatic disease in an HPV(−) patient.

**Figure 7:**
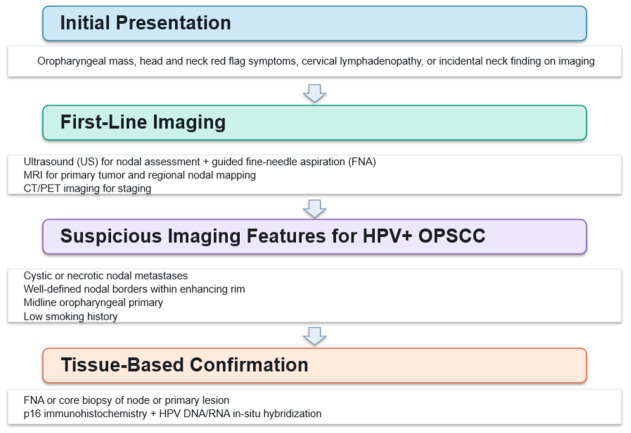
Proposed diagnostic pathway for oropharyngeal squamous cell carcinoma (OPSCC) with suspected HPV(+) status.

**Table 1:** Radiological findings using different imaging modalities, based on the literature review (main text).

	Radiological Findings in OPSCC Patients	
Imaging Modality	HPV(+)	HPV(−)
**Ultrasound**	No specific characterisation	No specific characterisation
**CT**	Cystic lymph node metastases Well defined borders of primary tumour Increased prevalence of nodal metastases Increased prevalence of extra capsular spread	Ill-defined borders of primary tumour More likely to invade adjacent muscles
**MRI**	Lower apparent diffusion coefficient of tumours	Not specified
**PET-CT**	Lower heterogeneity in primary tumour Higher heterogeneity in lymph node metastases Entropy higher in primary tumour	Higher heterogeneity in primary tumour Primary lesion larger (higher median total volume)
**Radiomics (on CT scans)**	Solid enhancing tumour Well delineated	Locally more aggressive Invasion of surrounding structures (e.g., muscles, pre-vertebral fascia) Fat blurring Extension to other anatomical regions

**Table 2:** Conflicting findings on tumour heterogeneity and related quantitative imaging features in HPV-positive and HPV-negative OPSCC.

Study	Imaging Modality	Site Assessed	Main Finding	Area of Uncertainty/Contradiction
**Cheng et al.** [27]	FDG PET-CT	Primary tumour	HPV(+) tumours showed lower uniformity and higher entropy, suggesting greater heterogeneity	Contrasts with studies reporting HPV(−) primaries as more heterogeneous; may reflect different heterogeneity metrics
**Tahari et al.** [28]	FDG PET-CT	Primary tumour and lymph nodes	HPV(−) primary tumours were more heterogeneous, whereas HPV(+) lymph nodes were more heterogeneous, likely due to cystic change	Differs from Cheng et al.; also highlights that findings depend on whether primary tumour or nodal disease is assessed
**Sharma et al.** [29]	FDG PET-CT	Primary tumour and lymph nodes	HPV(+) patients showed greater homogeneity in the primary lesion relative to corresponding lymph nodes than HPV(−) patients	Adds further complexity by comparing heterogeneity within the same patient rather than between tumour groups alone
**Tahari et al.** [28]	FDG PET-CT	Primary tumour	HPV(−) primary tumours were significantly larger than HPV(+) tumours	Contradicted by Sharma et al., who found no significant MTV difference by HPV status
**Yu et al./Altinok et al.** [49,52]	CT radiomics	Primary tumour	HPV(+) tumours appeared more compact and homogeneous, whereas HPV(−) tumours were more irregular and heterogeneous	Broadly aligns with Tahari et al. for primary tumours, but contrasts with Cheng et al., again suggesting metric- and modality-dependent heterogeneity findings
**Zwirner et al.** [73]	Radiogenomics	Primary tumour	FAT1 mutation, seen predominantly in HPV(−) tumours, was associated with reduced radiomic intratumor heterogeneity and better survival	Suggests that molecular subtype may influence heterogeneity independently of HPV status alone
**Clasen et al.** [74]	PET-MRI radiogenomics	Spatially sampled primary tumour regions	No conclusive association between imaging heterogeneity and genetic heterogeneity was identified	Contrasts with radiogenomic studies suggesting that imaging heterogeneity may reflect underlying molecular biology
**Nakahira et al., Driessen et al., Vangel et al. [36,37,38,39]**	MRI	Primary tumour	HPV(+) primary tumours tended to have lower ADC values than HPV(−) tumours	Interpretation is complicated because HPV(+) nodal metastases are often cystic and may show higher ADC, making comparisons context dependent
**Nakahira et al. [36]; Driessen et al. [37]; Vangel et al. [36,37,38,39]**	MRI	Primary tumour vs. nodal metastases	Lower ADC in HPV(+) primary tumours but potentially higher ADC in HPV(+) cystic nodes	Represents an apparent contradiction driven by anatomical site and cystic versus solid tumour composition rather than true disagreement

## Data Availability

No new data were created or analyzed in this study. Data sharing is not applicable to this article.

## Supplementary Materials

The following supporting information can be downloaded at: https://www.mdpi.com/article/10.3390/cancers18101648/s1, Figure S1: PRISMA Search.

## Abbreviations

The following abbreviations are used in this manuscript:

ADC	Apparent Diffusion Coefficient
AJCC	American Joint Committee on Cancer
AI	Artificial Intelligence
AUC	Area Under the Receiver Operating Characteristic Curve
CT	Computed Tomography
CpG	Cytosine–Phosphate–Guanine
DL	Deep Learning
DNA	Deoxyribonucleic Acid
FDG	Fluorodeoxyglucose
HIF-1α	Hypoxia-Inducible Factor 1 Alpha
HNSCC	Head and Neck Squamous Cell Carcinoma
HPV	Human Papillomavirus
HU	Hounsfield Unit
MRI	Magnetic Resonance Imaging
MTV	Metabolic Tumour Volume
OPSCC	Oropharyngeal Squamous Cell Carcinoma
PET-CT	Positron Emission Tomography–Computed Tomography
SUV	Standard Uptake Value
TCGA	The Cancer Genome Atlas
US	Ultrasound
