# Supplementary figures and images for: Radiological Features of Human Papillomavirus (HPV)-Positive and HPV-Negative Oropharyngeal Squamous Cell Carcinoma (OPSCC)—Considerations for Multimodal Analysis
Source: Cancers (Basel). 2026 May 20;18(10):1648. doi: 10.3390/cancers18101648 (PMC13204047; doi:10.3390/cancers18101648)

## Supplementary Materials:

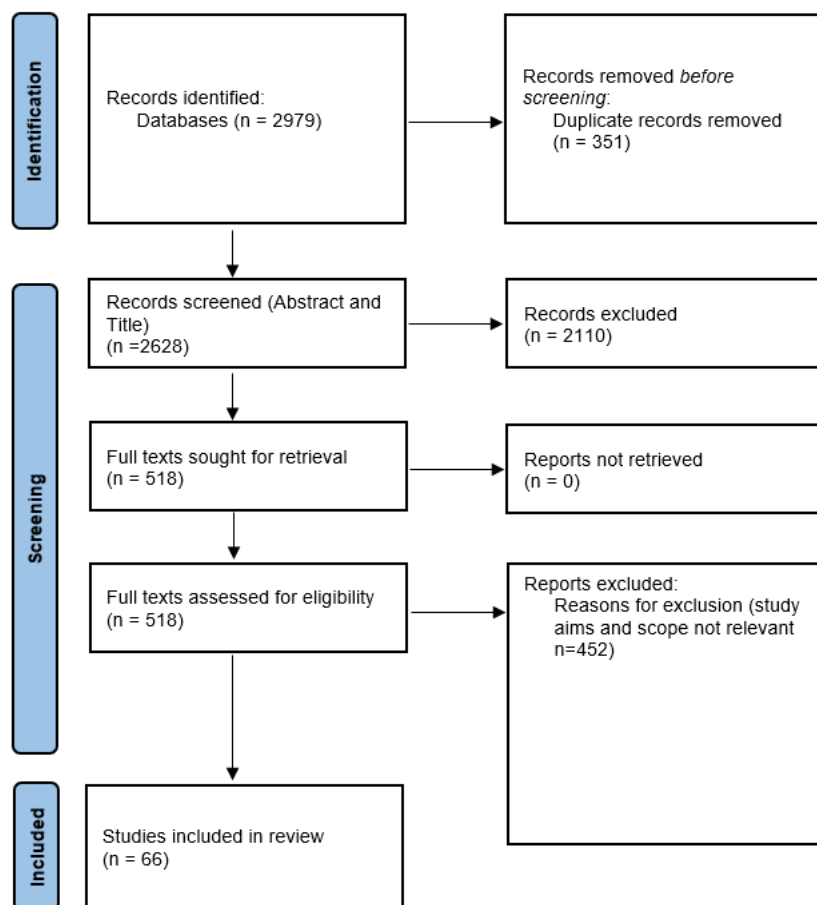

**Supplemental Figure S1. PRISMA Search.**

Supplement: Supplementary file 1 [file cancers-18-01648-s001.zip › cancers-4323853-supplementary.pdf]
